# Analysis of the evolution of COVID-19 disease understanding through temporal knowledge graphs

**DOI:** 10.3389/frma.2023.1204801

**Published:** 2023-08-03

**Authors:** Alessandro Negro, Fabio Montagna, Michael N. Teng, Tempestt Neal, Sylvia Thomas, Sayde King, Ridita Khan

**Affiliations:** ^1^Graph Aware Ltd., London, United Kingdom; ^2^Division of Allergy and Immunology, Department of Internal Medicine, University of South Florida Morsani College of Medicine, Tampa, FL, United States; ^3^Cyber Identity and Behavior Research Laboratory, Department of Computer Science and Engineering, College of Engineering, University of South Florida, Tampa, FL, United States; ^4^Advanced Membrane and Materials Bio and Integration Research Laboratory, Department of Electrical Engineering, College of Engineering, University of South Florida, Tampa, FL, United States

**Keywords:** COVID-19, temporal graph analysis, knowledge graphs, keywords, unstructured data, knowledge representation, keywords analysis

## Abstract

The COVID-19 pandemic highlighted two critical barriers hindering rapid response to novel pathogens. These include inefficient use of existing biological knowledge about treatments, compounds, gene interactions, proteins, etc. to fight new diseases, and the lack of assimilation and analysis of the fast-growing knowledge about new diseases to quickly develop new treatments, vaccines, and compounds. Overcoming these critical challenges has the potential to revolutionize global preparedness for future pandemics. Accordingly, this article introduces a novel knowledge graph application that functions as both a repository of life science knowledge and an analytics platform capable of extracting time-sensitive insights to uncover evolving disease dynamics and, importantly, researchers' evolving understanding. Specifically, we demonstrate how to extract time-bounded key concepts, also leveraging existing ontologies, from evolving scholarly articles to create a single temporal connected source of truth specifically related to COVID-19. By doing so, current knowledge can be promptly accessed by both humans and machines, from which further understanding of disease outbreaks can be derived. We present key findings from the temporal analysis, applied to a subset of the resulting knowledge graph known as the temporal keywords knowledge graph, and delve into the detailed capabilities provided by this innovative approach.

## 1. Introduction

The COVID-19 pandemic revealed the critical need for rapidly understanding the nature of any infectious disease before its outbreak reaches a critical state of community spread. Studies of previous infectious outbreaks show that public adherence to public health guidelines is greater when the scientific knowledge base surrounding the disease is stronger (Bults et al., 2011; Lin et al., 2011). The ability of global scientific leadership to communicate to the public with certainty surrounding risks, symptoms, and prevention is critical. For example, with respect to the U.S. population in response to COVID-19, research shows that knowledge levels within the public are related to the likelihood of an individual engaging in preventative measures and complying with public health guidelines (Clements, 2020). While there has been some research focused on the dissemination of information to the public via social media (Chan et al., 2020), there has been far less focus on enhancing the rate at which scientific information surrounding COVID-19 is aggregated and mined to advance the knowledge base. Such knowledge is critical for coherent and consistent information sharing.

Currently, science surrounding new diseases moves at the pace of scientists' current knowledge and their ability to read, digest, and synthesize information across multiple scholarly articles, and then utilizing this knowledge and expertise to find points of integration across the concepts and results. The potential for natural language processing (NLP), in which automated techniques can mine scholarly content, as a means of achieving these same outcomes has been previously outlined (Hirschberg and Manning, 2015), even in the context of knowledge graphs construction (Luan et al., 2018). Nevertheless, little has been done to pair NLP with network science and temporal analysis to connect key findings and concepts and synthesize their evolving nature over time. In simpler terms, it is crucial to merge new discoveries with well-established practices into a unified temporal knowledge repository. This integrated source serves as a reliable foundation from which relevant knowledge can be distilled, results can be validated, trends can be identified, and new findings can be continually shared in an iterative process. As such, this article moves from this idea covering three important aspects:

1. Gathering and organizing fast-growing and heterogenous knowledge in a single connected source of truth that is easy to access for humans and machines, specifically considering the temporal aspects in the graph modeling;
2. Identifying automated techniques to perform meaningful temporal analysis of the resulting knowledge base; and
3. Tracing the evolution of knowledge in a formal way, identifying patterns, and recognizing early-stage trends.

Achieving these three outcomes can improve the handling of similar infectious diseases by the identification of common static and dynamic patterns, providing just-in-time information, and accelerating the search and the navigation of an enormous amount of information. As such, the key element of this effort is the presentation of a novel application of knowledge graphs for disease understanding, which both aggregates current evolving science with pre-existing knowledge bases and allows temporal exploration of this information.

While many sources are used by professionals to find treatments, approaches, and the latest discoveries, these are dispersed, heterogenous, difficult to search, or disconnected. As a result, discoveries around COVID-19 cannot be analyzed in a proper way, and already adopted therapies cannot be easily discovered. In the early stages of the COVID-19 outbreak, many researchers (Michel et al., 2020; Chen et al., 2021) focused on the gathering of knowledge from literature and organize it in the form of knowledge graph, mostly in the Resource Description Framework format, making it available to downstream applications. This approach showed the value of the knowledge graph in gathering information from multiple sources, prompting others to explore similar approaches for various purposes. For example, to enhance search capabilities over the expanding literature, Wise et al. (2020) built a COVID-19 knowledge graph to extract complex relationships between related scientific articles. Their goal was to implement an advanced search engine to assist researchers and policymakers in extracting timely information to address key scientific questions about COVID-19 from a corpus of scientific articles. Similarly, Wang Q. et al. (2020) constructed a knowledge graph to aid clinicians in analyzing COVID-19-related information and tackling complex tasks like drug repurposing. Leveraging existing knowledge bases, Cernile et al. (2021) also built a knowledge graph from scientific publications related to COVID-19, using CORD-19 (Wang L. et al., 2020) as a data source. Their work demonstrated how knowledge graphs enable rapid navigation and exploration of inter-relationships among entities, improving the understanding of diseases such as COVID-19.

In a similar vein, our approach centers on utilizing a knowledge graph to consolidate the literature related to COVID-19. However, our primary focus diverges from previous studies as we emphasize exploring the temporal evolution of our understanding of COVID-19. Specifically, our key objective is to develop a framework that effectively captures the evolving nature of knowledge over time. This unique objective introduces certain peculiarities into the graph model, ultimately enabling distinctive analyses. To achieve this, we build upon existing methodologies of knowledge graph creation. For example, our pipeline to develop a temporal knowledge graph follows an iterative and incremental lifecycle, based on an existing Linked Data lifecycle model that has been already applied in real-world scenarios (Hyland and Wood, 2011; Villazón-Terrazas et al., 2011), and incorporate existing techniques such as time slicing (Choudhury et al., 2020). By leveraging these established methods, we ensure the meaningful utilization of prior scientific advancements to ultimately convert multiple and heterogeneous data sources, some of which are unstructured, into a single connected source of truth related to the COVID-19. Leveraging temporal information, we slice the graph into multiple time-bounded sub-knowledge graphs. As a result, our approach presents a novel use case for knowledge graphs, particularly in mapping the changes in specific topics and their relevance as the COVID-19 disease progresses. Through this innovative approach, we shed light on the dynamic nature of knowledge within the context of COVID-19. It is worth noting that the analyses performed on our graph showcase different algorithms for information aggregation and extraction, which can be applied to other diseases as well.

## 2. Methodology

Knowledge graphs (KGs) have emerged as a core abstraction for incorporating knowledge into intelligent systems (Hogan et al., 2021). KGs can be generally described as an “evolving graph data structure, composed by a set of typed entities, their attribute and meaningful named relationships among them, built for a specific domain with the intent to craft knowledge for humans and machines” (Negro et al., 2023). Thus, a KG represents a specific domain of knowledge by means of entities and relationships in a graph structure. KGs are easily accessible for both humans and machines to augment their capabilities and are flexible to enable a continuous manipulation and ingestion of various data from different data sources. Moreover, the materialization, storage, and access to the information included in a KG efficiently supports offline analysis and online visualization and processing. Given these capabilities, KGs are a powerful tool for modeling the relations between entities in various fields, from biotechnology to e-commerce, intelligence, law enforcement, and financial technology (Szekely et al., 2015; Liu et al., 2019; Li et al., 2020, 2022; Xu et al., 2020; Feng et al., 2022), among diverse language and text-based applications, including search engines, chatbots, and recommendation systems (Liu et al., 2020; Zhou et al., 2020).

There is at least a two-fold perspective that characterizes KGs. The first perspective focuses on knowledge representation, in which the graph is encoded as a collection of statements formalized using the Resource Description Framework (RDF) data model (Govindapillai et al., 2021). Its goal is to standardize data publication and sharing on the Web, ensuring semantic interoperability. In the RDF domain, the core of intelligent systems is based on the reasoning performed on the semantic layer of the available statements. The second perspective focuses on the structure (properties and relationships) of the graph. This vision is implemented in the so-called Labeled Property Graph (LPG) (Purohit et al., 2021). It emphasizes the features of the graph data, enabling new opportunities in terms of data analysis, visualization, and development of graph-powered machine learning systems to infer further information. Leveraging these advances, KGs can help researchers tackle many biomedical problems, such as finding new treatments for existing drugs (Himmelstein et al., 2017), aiding efforts to diagnose patients (Choi et al., 2017), and identifying associations between diseases and biomolecules (Shen et al., 2017).

### 2.1. Knowledge graph construction with the linked data lifecycle

KGs are generally constructed using the Linked Data lifecycle. This lifecycle includes specification, modeling, data lifting, data publication, and data curation for “publishing and connecting structured data on the Web” (Ngomo et al., 2014). The specification consists of the identification of main goals, requirements, and constraints that drive the features and shape of the final model, along with the data that will be integrated within the KG. Modeling involves identifying key entity classes and the relationships among them, along with the vocabulary that specifies the set of allowed instances of interest. Data lifting, or data ingestion, refers to the ingestion of data, leading to the final KG. This involves transforming both structured and unstructured data from the original schema to the target schema and linking entities from multiple sources together. In some cases, the schema requires two entities coming from different sources to be merged in a single node of the graph. Data publication makes the KG accessible, such as through a standard API, a generic frontend, or a graph visualization tool. Finally, data curation cleans, maintains, and preserves data for reuse over time.

### 2.2. Data sources, modeling, and the schema

The effectiveness of any analysis heavily relies on the quality of the input data. Therefore, prior to delving into the temporal exploration of COVID-19, our initial focus was on constructing a robust KG that would serve as a solid foundation for our analysis. Thus, we gathered several sources of information concerning SARS-CoV-2 and COVID-19, along with pre-existing relevant data, to conduct a comprehensive analysis of the evolving understanding and critical aspects of a novel disease outbreak. By ensuring the completeness and accuracy of our KG, we established a reliable basis for subsequent analyses.

Since the early stages of the spread of this disease, diverse information sources have been publicly available (e.g., Wahltinez et al., 2022; Centers for Disease Control and Prevention, n.d.) with the specific intent to accelerate the knowledge distribution and learning curve around the disease. In addition, other knowledge sources were already available to professionals in digital format to feed different autonomous intelligent systems. Thus, the data sources used in this project are completely publicly available. They include the following:

- Hetionet (Himmelstein et al., 2017) is a network of biomedical knowledge assembled from 29 different databases of genes, compounds, diseases, and more.
- Uniprot (Bateman et al., 2022) is a freely accessible resource of protein sequence and functional information.
- CORD-19 (Wang L. et al., 2020) is a resource with over 200,000 scholarly articles on COVID-19, SARS-CoV-2, and related coronaviruses.
- Drug Repurposing Knowledge Graph (DRKG) (Ioannidis et al., 2020) is a comprehensive biological KG relating genes, compounds, diseases, biological processes, side effects, and symptoms. It includes information from six existing databases including DrugBank, Hetionet, GNBR, String, IntAct, and DGIdb, and data collected from recent publications particularly related to COVID-19.
- Gene Ontology (GO) (Gene Ontology Consortium, 2004) is the world's largest source of information on the functions of genes. This knowledge is both human-readable and machine-readable and is a foundation for computational analysis of large-scale molecular biology and genetics experiments in biomedical research.
- Medical Subject Headings (MeSH) (Lipscomb, 2000) is the National Library of Medicine's controlled vocabulary thesaurus used for indexing articles for PubMed.

**Figure 1:**
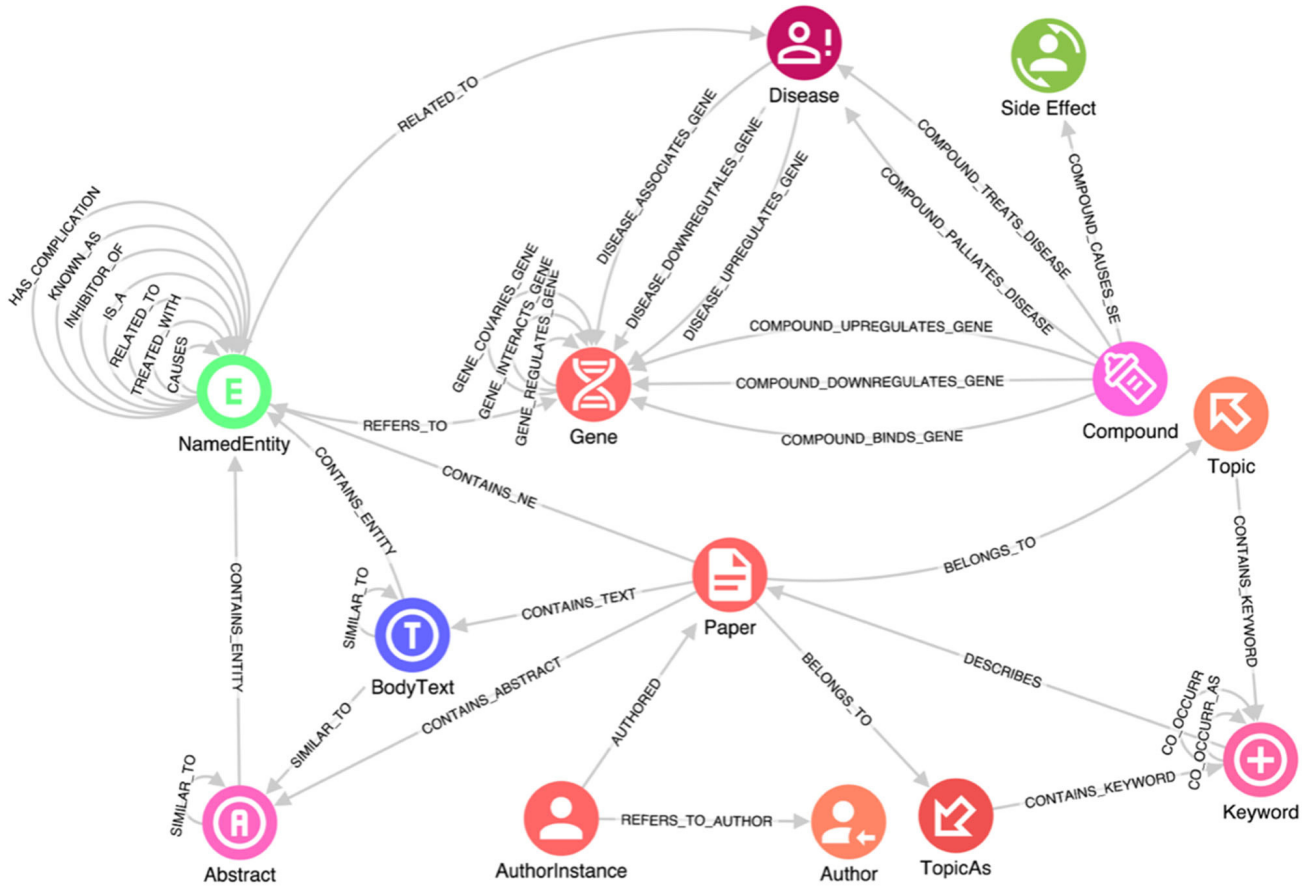
A portion of the knowledge graph schema. This schema captures data about genes, diseases, compounds, and side effects, along with their interactions, e.g., how a disease is connected to a specific gene, how it can be treated by a specific compound, and the side effects of such compound. Research manuscripts are connected from one author to another author by institution, and relevant relationships between manuscript sections are retained.

When working with structured data, importing it into a KG is relatively straightforward. For instance, the Hetionet database is already structured as a graph of nodes and relationships, conveniently provided in two .csv files—one containing all the nodes and the other containing all the relationships. On the other hand, unstructured free text lacks explicit structure, which makes it challenging to search for and analyze the information contained within (Grishman, 2015). Extracting and processing such structures are main tasks in NLP. Specifically, information extraction (IE) is a key step in making a text's semantic structure explicit, and thus, useful. More precisely, IE is the process of analyzing text to identify semantically defined entities and relationships. Further, recognizing relevant entities and the relationships among them are critically relevant intermediate steps. In the case of this study, these entities include genes, proteins, symptoms, compounds and so on, and their relationships. The task of named entity recognition (NER) involves finding each mention of a named entity in the text and labeling its type (Grishman and Sundheim, 1996). Note that an entity can also be composed of multiple tokens extracted; the same would happen for our domain, where severe acute respiratory syndrome (SARS) must be considered as a single entity. Moreover, the recognized entities are connected on one side to the source paper containing them and on the other to the reference knowledge bases (e.g., Hetionet, Uniport, etc.). In Figure 1, the class NamedEntity in the schema results from the IE process.

In addition, due to COVID-19 being a novel disease with new relationships to existing genes, proteins, and other relevant elements, determining the most likely diagnosis based on symptoms requires not only the identification of specific entities but also the understanding of the connections between them. These relationships are expressed within the text data through specific sentences in which researchers mention them. Therefore, it is essential to enrich the information in the reference knowledge bases with new relationships inferred from the text using Entity Relationship Extraction (ERE) techniques. This process allows us to extract relevant relationships from the text and incorporate them into the KG, thereby enhancing its completeness and capturing the evolving understanding of COVID-19. One common algorithm used for relation extraction is based on lexico-syntactic patterns (Negro, 2021). This algorithm involves mapping syntactic relationships among tokens or specific sequences of tags to a set of relevant relations between key named entities. By applying a series of semantic analysis rules, each designed to map a subgraph of the syntactic graph (a portion of the graph containing syntactic relationships that connect key entities), anchored by mentions of certain entities, we can associate them with corresponding relations in the database. This approach provides a rough yet effective approximation. ERE plays a significant role in improving the quality of a KG in terms of the insights extracted and the available access patterns. By applying ERE techniques, connections are created between the NamedEntity entries extracted from the text, enabling seamless navigation and exploration of the graph. This facilitates the production of a meaningful and informative graph that captures the evolving understanding of COVID-19 and enhances the insights that can be derived from it. In Figure 1, these relationships are represented by self-connections on the NamedEntity class.

After several iterations of the Linked Data lifecycle, a reasonable schema for exploration and analysis was derived using the data sources listed above. The full schema is complex; a subset is provided in Figure 1. This schema captures data about genes, diseases, compounds, and side effects, along with their interactions, e.g., how a disease is connected to a specific gene, how it can be treated by a specific compound, and the side effects of such compound, from structured and unstructured data sources. Research manuscripts are also connected from one author to another author by institution, and relevant relationships between manuscript sections are retained. The full import process of data is accomplished using GraphAware Hume Orchestra—the workflow engine available in GraphAware Hume. Specifically, GraphAware Hume^7^ was used as the main tool for data gathering, merging and transformation as well as analytics and graph visualization. It provides facilities for data orchestration, including support for unstructured data, and many different algorithms for analysis and graph visualization for knowledge exploration.

### 2.3. Extending knowledge graphs for temporal analysis

The KG presented in Section 2.2 encompasses a wide range of information, making it suitable for effective representation within a temporal framework. Our approach primarily focuses on research papers, authors, and keywords as the basis of analysis within the KG. Each paper in the KG includes temporal information derived from its publication date. By leveraging this temporal dimension, we can map it onto a specific portion of the graph and incorporate time as attributes within relationships. This enables the creation of a dynamic co-occurrence graph of keywords, providing valuable insights into the evolving landscape of COVID-19 research over time.

To evaluate our approach, we chose to use keywords as they offer a concise expression of authors' understanding, thematic context, and research summaries. Moreover, keywords are commonly used for indexing purposes in digital libraries, making them powerful tools for knowledge discovery (Song et al., 2013). The resulting time-reach co-occurrence graph, which we refer to as the “TagGraph” for simplicity, is isolated and utilized for temporal analysis. Here, the term “tag” is preferred over “keywords” as it represents a more generic term, allowing for the potential application of our analysis to any textual element that can be attached to or automatically extracted from text.

Consequently, a key objective of our work is to facilitate the improved identification of research progress, common patterns, trends, and emerging anomalies. Once our approach is validated and consolidated, it may be possible to generalize it to other areas of the graph that exhibit temporal dynamics. Furthermore, in the future, our methodology could be applied to studying unknown diseases as they emerge.

#### 2.3.1. Approach

The temporal analysis of a TagGraph focuses on the evolution of the co-occurrence of author keywords, or tags, provided directly by a paper's authors to categorize the major contributions of their article. These author-selected tags are carriers of knowledge units, or knowledge entities (Su and Lee, 2010). The co-appearance of two author-selected tags in an article defines a certain relationship between two topics. Multiple such instances denote the strength of their relationships (Yang et al., 2011). The assumption is that two tags appearing in the same article imply that the concepts represented by these tags are correlated. The more authors that use the same pair of tags, the more related they are. In this section, we describe our approach for generating a TagGraph, an application of a generic KG, which leverages relationships between tags for extracting evolving knowledge about COVID-19.

Connections among paper topics are not static. Scientific knowledge creation is dynamic; different avenues of research converge, and new connections emerge among disjointed and existing areas of science (Pan et al., 2012). This knowledge is generally incremental besides a few revolutionary and fundamental changes. New hypotheses are being postulated by encompassing existing scientific concepts from multiple domains. Canals (2005) pointed out that the diffusion of scientific knowledge can be mapped into a network structure where knowledge propagates via interactions among networked agents, in our case, the authors. Thus, a TagGraph is a temporal-bounded co-occurrence graph where nodes are tags, or keywords, and edges represent their causal relationships over the time. In addition to causal relationships, statistically significant and non-trivial co-occurrence patterns of tags also represent their semantic affinity (Montemurro and Zanette, 2013) and relatedness (Schulz et al., 2014).

**Figure 2:**
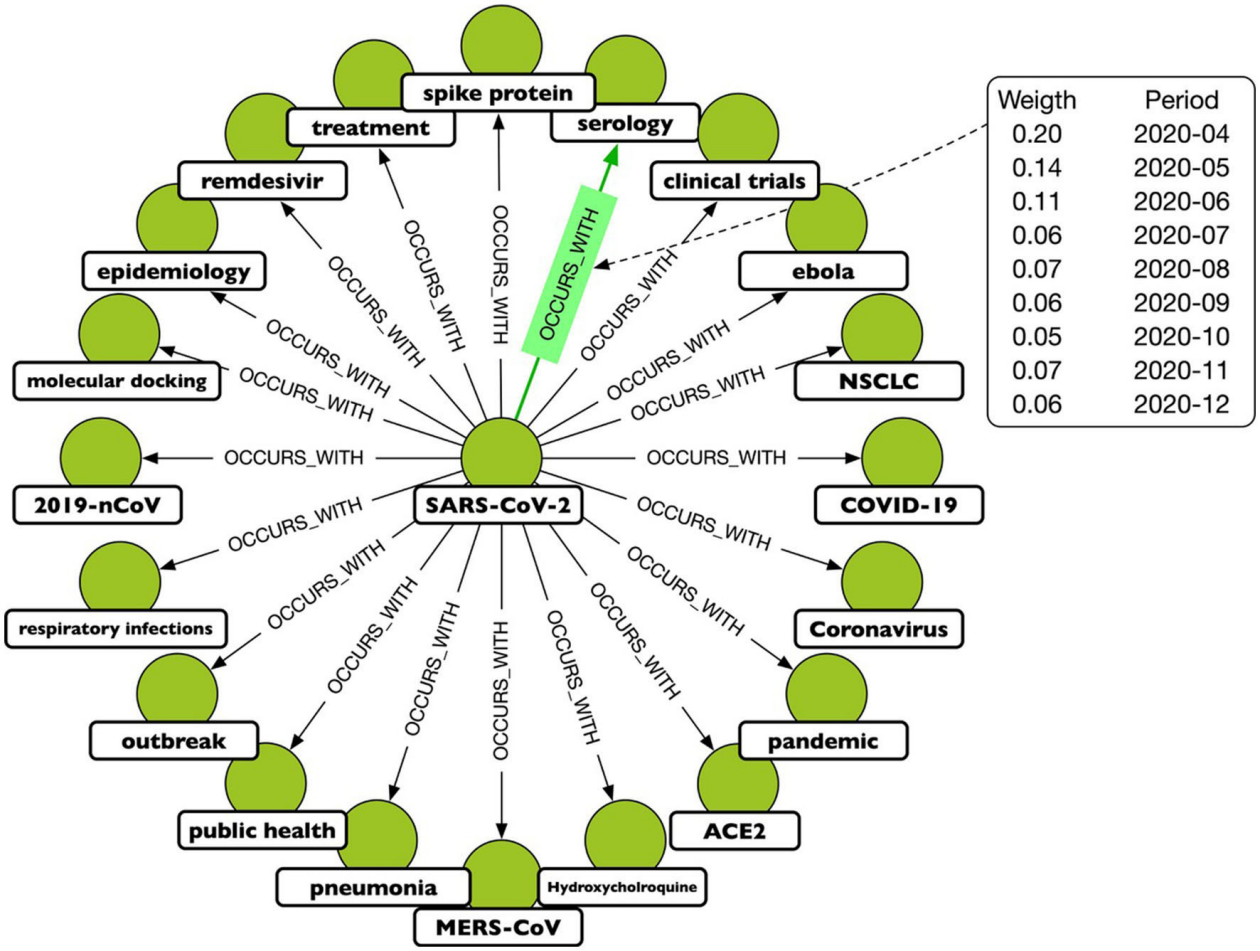
A portion of the projected graph, where the OCCURS_WITH relationship connects keys that have been mentioned in the same paper. The highlighted relationship shows that the keys “SARSCoV-2” and “serology” appear often together, although with varying frequencies over the period under consideration. As a result, each relationship is associated with a weight that is a function of time.

A TagGraph's analysis is dynamic by the creation of multiple “temporal snapshots” of a TagGraph by month. That is, a temporal snapshot is a network *G*_*t*_ = (*V*_*t*_, *E*_*t*_) for the time *t* = 1, 2, …*T*, where *V*_*t*_ is the set of tags appearing in papers dated at time *t* and *E*_*t*_ is the set of relationships based on those papers. The vertices and the edges at time *t* can be new or recurring. The dynamicity of tag co-occurrences denotes that new research topics, hypotheses, or directions emerge over time through co-appearances of existing tags. In terms of modeling, this has been translated in a temporal relationship, OCCURS_WITH, where the temporal information is an added property. An example is depicted in Figure 2.

**Figure 3:**
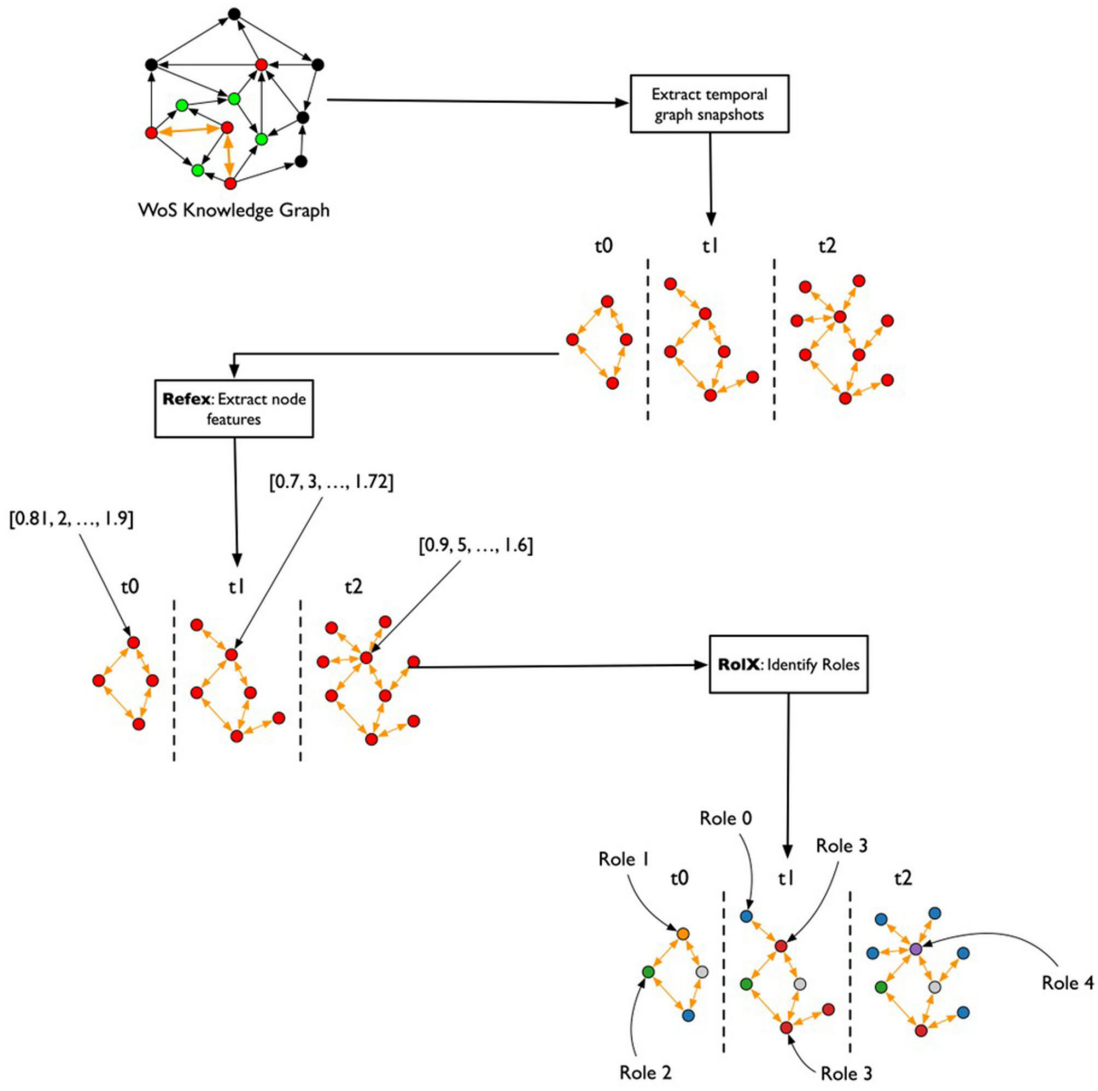
Flowchart for the temporal graph analysis. From the heterogeneous graph that represents the initial knowledge graph, monthly snapshots are extracted, describing the co-occurrence of keywords in papers published each month. The REFEX feature extraction algorithm is applied to each snapshot, associating each keyword with a different feature vector in each snapshot. The features are then aggregated and processed using the RolX algorithm, which assigns a role to each keyword for each month.

After the creation of the TagGraph temporal snapshots, the analysis leverages Role-Dynamics (Rossi et al., 2012), which further leverages ReFeX (Henderson et al., 2011) and RolX (Henderson et al., 2012) algorithms. ReFeX characterizes each node by structural graph features, while RolX performs matrix factorization over the nodes features matrix to identify “roles” of nodes in the graph, or nodes that have similar structural features. The target of the Role-Dynamics approach is to analyze how such roles evolve over time, which we evaluated from March 2020 to March 2022. As shown in Figure 3, the general workflow for analyzing temporal changes consisted of extracting the temporal co-occurrence graphs; running ReFex on all the nodes for all snapshots to extract the most relevant structural features for each; normalizing the ReFeX features between 0 and 1 to improve the results of the next phase; and running RolX over the full time-series. The output of this process is the definition of a small set of roles that effectively describe the node behaviors in a time-consistent way and the characterization of each node as a temporal mixture of such roles.

#### 2.3.2. Graph projection and temporal discretization

Due to the arbitrariness with which authors choose their tags, including misspelling, mixing acronyms, etc., the overlap of tags for the same concept is heavily reduced. This affects the quality and structure of the temporal snapshots and the consequent results of the entire process. To mitigate this issue, tags are associated using a combination of sentence embedding (to vectorize the tags in a latent space) and a clustering algorithm to create groups of tags with the same meaning. The SPECTER Bert model (Cohan et al., 2020) is used for the embeddings and DBSCAN (Ester et al., 1996) for the clustering. The approach of combining those techniques for merging and cleaning the tags represents another novelty of this work and it improves the quality and stability of the results. The TagGraph is then computed at the cluster level with the same approach described in Section 2.3.1, computing it in monthly snapshots. Thus, hereafter, a “tag” that represents this cluster of tags.

As previously stated, the snapshots are computed by month to have appropriate granularity and reveal early patterns. There are different techniques for measuring the strength of this association. We used the formula of the association strength (Eck and Waltman, 2009):


$$\begin{array}{l} S_{A}\left(c_{ij},s_{i},s_{j}\right)=\frac{c_{ij}}{s_{i}s_{j}} \end{array}$$

where *c*_*ij*_ represents how many articles have both tags, while *s*_*i*_ and *s*_*j*_ represent the frequency of tags *i* and *j*, respectively. Based on this formula, we consider the relationship undirected since both directions have the same weight.

#### 2.3.3. Feature and role extraction

**Figure 4:**
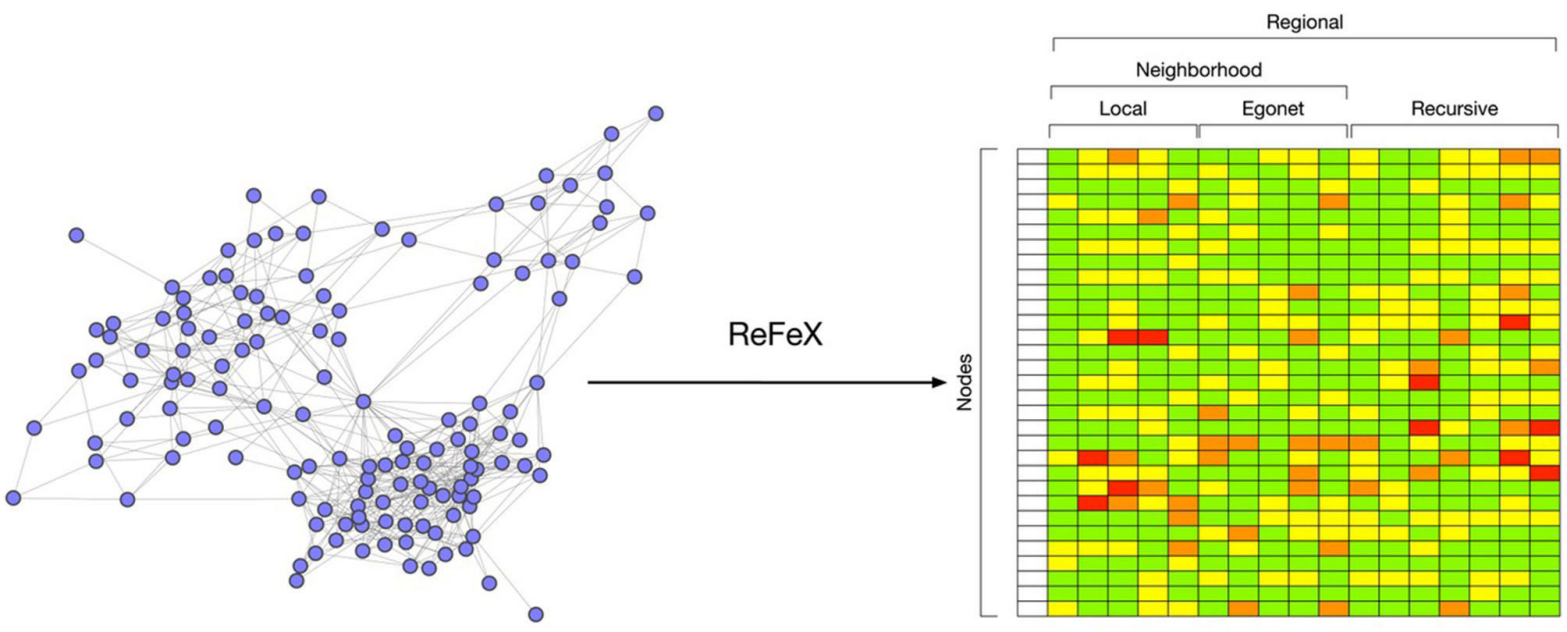
Conversion of each node into a vector representing the node's topological feature at different scales using ReFeX (Henderson et al., 2011).

The ReFeX algorithm is run over the monthly TagGraph snapshots. ReFeX is a structural graph feature that extracts base features at the node level to describe the statistics of each node neighborhood, aggregating these statistics recursively. Node level features include node degree, ego-net degree, page rank, eigenvector centrality, etc. The aggregation includes sums and means. The feature vector associated to each node is then composed by base features like *degree* which is a node scale property, and *degree(sum)*, which represents the sum of the *degree* property of the neighborhood for this node. The recursivity of the aggregation process makes it possible to compute features like *degree(sum)(mean)(mean)(sum)*, which aggregates information at a regional scale (Figure 4). The algorithm prunes irrelevant features at each iteration to avoid the exponential growth of the feature vector size.

**Figure 5:**
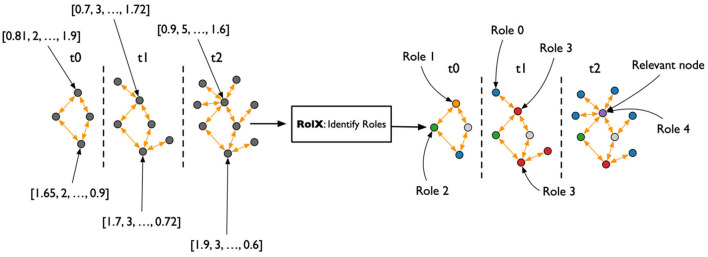
Compression of ReFeX feature vectors into smaller role vectors using RolX.

The output of the ReFeX algorithm is a tabular representation of the behavioral features of the TagGraph through time, which captures the complexity of the behaviors hidden in the topology of the relationships between nodes. The RolX algorithm introduces the idea that there exists a set of roles that the nodes can play, and such roles are able to explain the complexity of the observed structural features. The algorithm computes the optimal number of roles and how each role is connected to the set of available features. RolX then generates a model able to convert a ReFeX feature vector associated to each node at each time step to a much smaller vector representing the role mixture for that node at that time-step. The RolX assumption is that, while behaviors are complex to describe, the absolute numbers of such behaviors are comparatively low. If true, it should be possible to achieve a significative dimensionality reduction for the feature space without compromising the richness of the ReFeX results, as shown in Figure 5.

## 3. Results

Our understanding of infections, transmissions, treatments, and testing has evolved significantly over the course of the COVID-19 pandemic. Roles and role transitions captured in the dynamics of the TagGraph provide an autonomous mechanism to reveal understandable patterns in knowledge evolution to facilitate navigation of a huge number of related papers. Such a mechanism can help model the evolution of science more broadly, for instance, in for the next disease outbreak.

### 3.1. Role interpretations

An initial goal of our approach is the interpretation of the meanings of roles. While roles are extracted via matrix factorization applied to the feature matrix produced by ReFeX, they are difficult to interpret due to ReFeX's automatic extraction which uses an optimization objective function. Nevertheless, to certain extent, it is possible to map them to some well-known graph and node structure information, like the well-known and easy-to-understand node measures of PageRank, betweenness centrality, closeness centrality, degree, and the local cluster coefficient. The PageRank algorithm measures the importance of each node within the graph based on the number of incoming relationships and the importance of the corresponding source nodes. Betweenness centrality detects the amount of influence a node has over the flow of information in a graph. Closeness centrality detects nodes that can spread information very efficiently through a graph. The degree centrality algorithm finds popular nodes within a graph as it measures the number of incoming or outgoing (or both) relationships from a node, depending on the orientation of a relationship projection. Last, the local clustering coefficient of a node describes the likelihood that its neighbors are also connected.

These well-known measures are computed for each node in each snapshot. We used these results to build five matrixes, one for each role, where every row represents a node-snapshot pair. On the columns of these matrixes, we put the node relevance, i.e., the contribution of the matrix's role for the node and the snapshot of the row, and all the measures mentioned above. We used these matrixes to compute the pairwise correlation between the node relevance and every measure over all the node-snapshot rows. The results are presented in Table 1, with correlation values ranging between −1 and 1, where 1 means that the measure and the role relevance are directly correlated, −1 means that there is an inverse correlation, and 0 means no statistical correlation exists between the measure and the role relevance. Note that while we focused on these set of measures, it may be possible to extract additional measures that might help better define the roles.

**Table 1:** Roles-graph measures and correlations.

**Role**	**Graph Measure**	**Correlation**
Role 0	PageRank	0.843
	Betweenness	0.616
	Closeness	0.861
	Local Clustering	−0.410
	Degree	0.866
Role 1	PageRank	0.245
	Betweenness	0.061
	Centrality	−0.117
	Local Clustering	0.168
	Degree	0.286
Role 2	PageRank	0.691
	Betweenness	0.340
	Closeness	0.637
	Local Clustering	−0.823
	Degree	0.808
Role 3	PageRank	−0.607
	Betweenness	0.057
	Closeness	−0.323
	Local Clustering	0.350
	Degree	−0.694
Role 4	PageRank	−0.089
	Betweenness	−0.054
	Closeness	−0.389
	Local Clustering	−0.297
	Degree	−0.072

**Figure 6:**
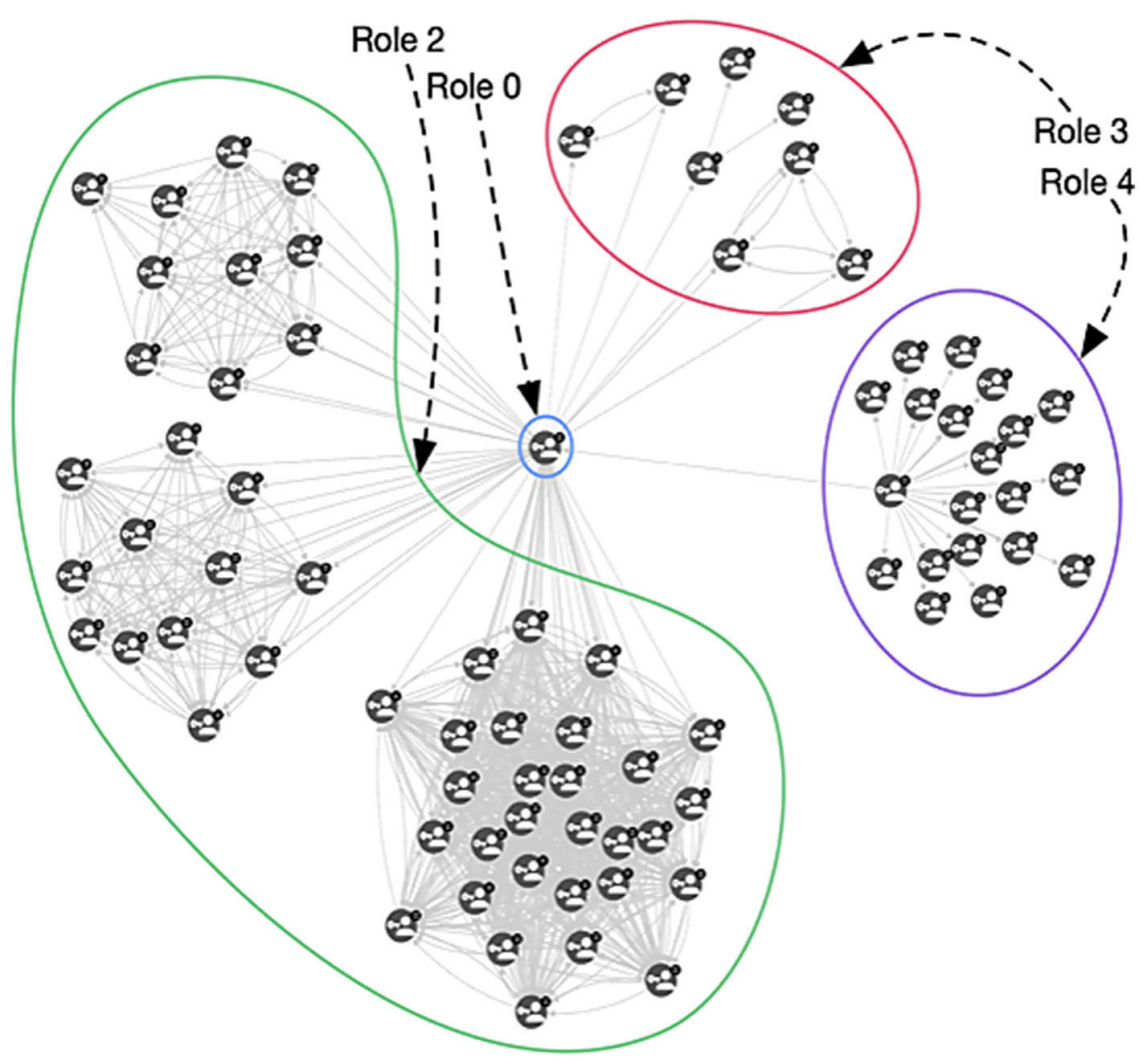
An example of a graph containing nodes with different role distribution. Role 0 and Role 1 nodes are strongly connected and central, Role 3 nodes are almost disconnected from the central nodes, and Role 4 nodes are structured such that they are peripheral but connected.

From Table 1, we can interpret some of the roles based on the correlation value. For example, Role 0 and Role 2 are directly related to all the measures we extracted, indicating that roles 0 and 2 identify nodes that are central in the network (related to the high value of betweenness and closeness centrality) and they are densely connected to other important nodes (related to the high correlation with PageRank and degree). Hence, these represent very important tags in that specific period. On the other hand, role 1 appears to be unrelated to any of the measures we computed. When analyzing the results of these tags, we noticed that they reflect noisy tags, i.e., tags that randomly appear in the network with no specific relevance of any type. The matrix factorization collected them under the same role 1. It is possible that uncomputed minor measures may better define this role. Role 3 is indirectly connected to PageRank, which means that the nodes having a high value of role 3 are not connected to any relevant node, and the indirect correlation with degree and closeness means that they are barely connected to anything. Thus, role 3 represents nodes that are on the edge of the co-occurrence network, and, in many cases, completely disconnected from it. Role 4 is similar to role 3, but since it is not as indirectly connected to degree value and PageRank as role 3, these nodes are not as isolated and are slightly connected to the rest of the network. These connections are not necessarily small, so these nodes could be connected to many of the nodes and some may be important. These relationships are depicted in Figure 6.

### 3.2. Story telling from temporal analysis of TagGraphs

**Figure 7:**
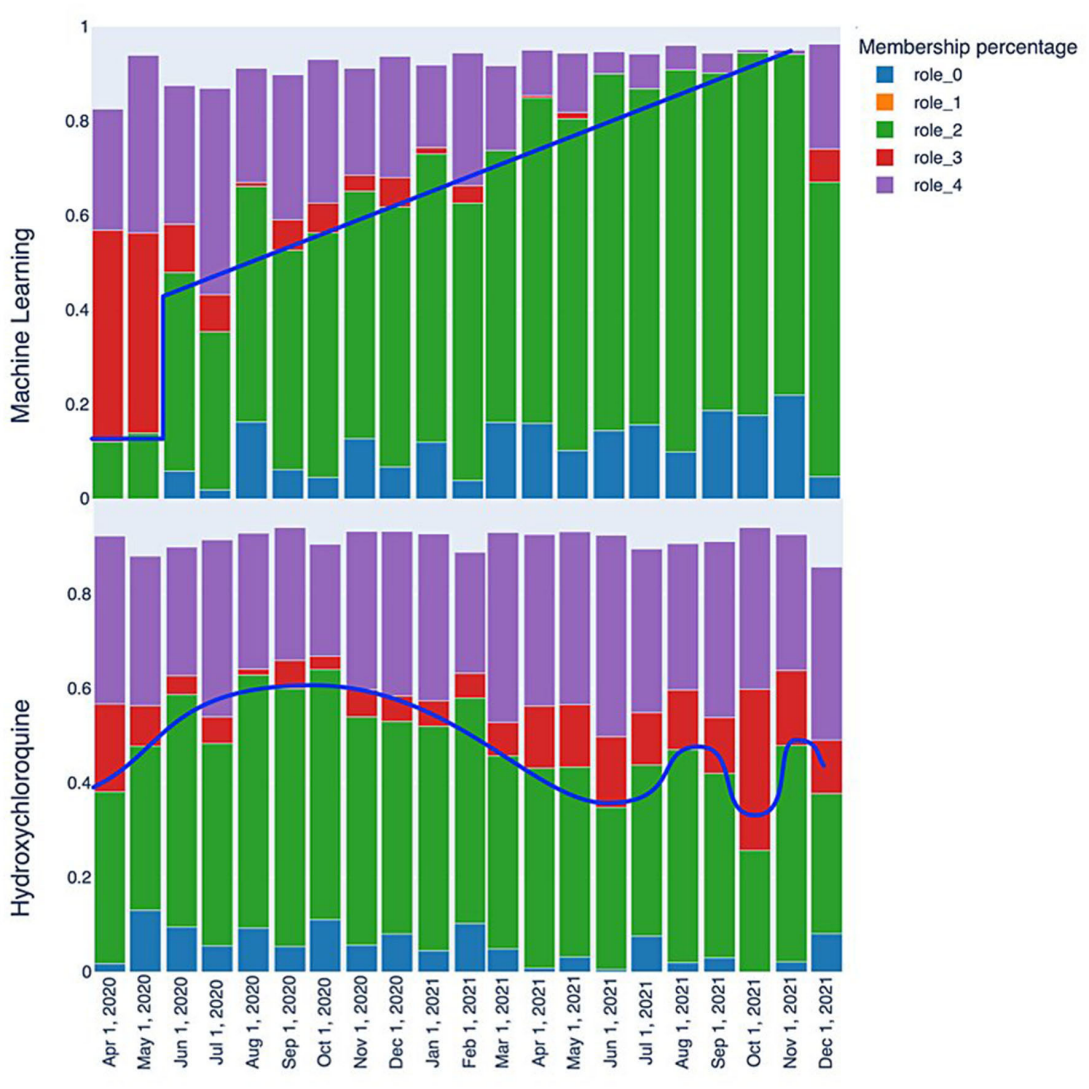
Role evolution comparison: the “machine learning” keyword quickly transitions from a marginal role to a relatively central one between May and June 2020, with a consistently positive trend that makes it a highly relevant keyword. On the other hand, the “Hydroxychloroquine” keyword displays fluctuating patterns that reflect the scientific community's interest in this molecule, with periods of higher and lower interest.

The initial analysis of the RolX results consisted of analyzing role evolutions through various snapshots for each of the tags since role interpretation is fundamental for understanding the dynamic graph evolution embodied in TagGraphs. The purpose of this inspection is to reveal patterns (similar behaviors in the transitions) and signals (clearly readable spikes or strong transitions among two or more snapshots) in the role's relevance bar chart. This analysis aims not only at identifying individual spikes or falls but also at revealing the speed of changes and similar types of patterns as shown in Figure 7. That is, we can clearly identify that “machine learning” has a steady progression in role 2 and role 0 over time as represented by the blue line. Another interesting tag, revealed by the analysis of roles evolution, is “hydroxychloroquine,” a drug used to treat certain autoimmune diseases that were shown to have antiviral activity against SARS-CoV-2 in specific cell lines although clinical trials showed no antiviral effect of hydroxychloroquine in people. Hence, after the initial enthusiasm, this drug has not been used as a SARS-CoV-2 antiviral. The related behavior is evident also in the role bar chart; the initial spike in roles 0 and 2 grows and then degrades over time. Despite some fluctuation, roles 0 and 2 end lower while role 3 increases, locating these studies at the margin of clinical research. This type of analysis focuses on single tags and, thus, could be used to identify patterns that can be then used to search for commonalities in other tags. While this approach is powerful since it can be easily automated once the signals have been identified, it suffers in the definition of a “story” around the data that is easier to understand and more stable across different data sources.

**Figure 8:**
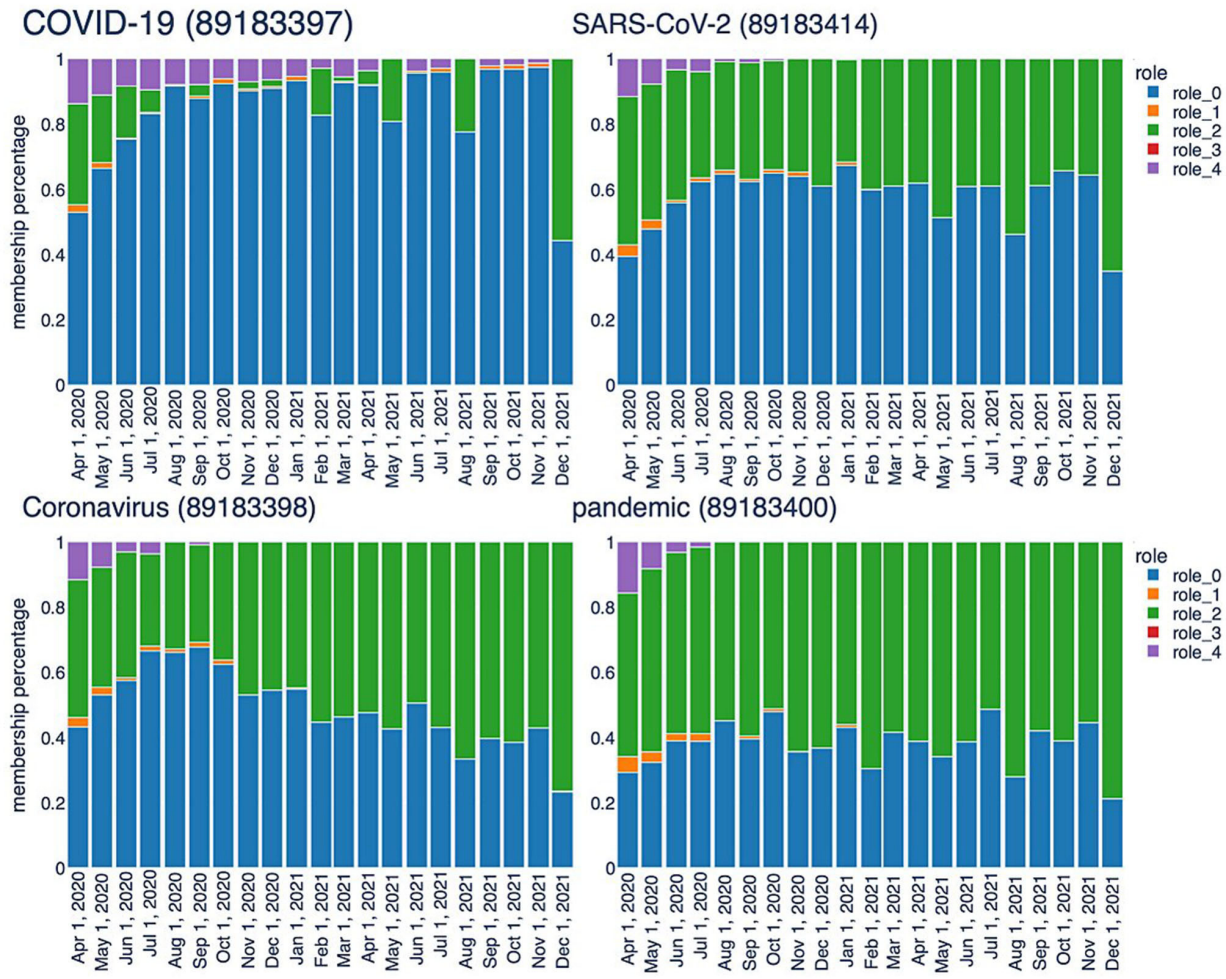
Temporal trend of central keys characterized by high values associated with Role 0 and Role 2.

The second type of analysis facilitated by the TagGraph structure combines neighborhood exploration with the roles extracted by RolX. Assisted by the role interpretations described above, we can generate more human-readable results. The analysis starts from a few tags that represent the center, i.e., the most relevant nodes in the co-occurrence network. By utilizing signal analysis, certain tags exhibit a clear and strong signal for role 0 and role 2, which remains consistent throughout the entire history sampling, including “SARS-CoV-2”, “COVID-19”, and “Coronavirus”. Their roles transition bar charts are represented in Figure 8. These tags are clearly key terms that represent the focus of the research articles we processed. Notably, the RolX transitions inspection reveals them autonomously, validating, once more, the hypothesis that the adopted approach can reveal such patterns. In our case, “SARS-CoV-2” represents a cluster of tags related to the virus, “COVID-19” contains the disease-related tags, and “Coronavirus” encapsulates the terms connected to the family of viruses related to SARS-CoV-2. The neighborhood analysis revolves around starting from the most significant tags for our target analysis, namely the three tags mentioned above, and identifying the most relevant tags connected to them. This search is performed for each snapshot, and the results are compared to extrapolate how understanding has evolved over time. Defining the most relevant tags poses a primary challenge. In this stage of analysis, we focused on examining each role in isolation and considered only the directly connected nodes, postponing the analysis of the egonet to a future iteration of our work. Specifically, node relevance is computed using the following formula:


$$\begin{array}{l} node\_relevance(start\_node,\;end\_node,\;role\_name,\;t)\\ \;=\;relationship\_weight(start\_node,\;end\_node,\\ \;t{)}^{*}role\_relevance(end\_node,\;role\_name,\;t) \end{array}$$

where:

- *start*_*node* is the center of the analysis, i.e., “SARS-CoV-2”, “COVID-19”, or “Coronavirus”.
- *end*_*node* is on the nodes belonging to *neighbour*(*start*_*node, t*), or all tags connected in the co-occurrent network of the time frame *t*.
- *relationship*_*weight* is the weight of the relationship connecting *start*_*node* and *end*_*node* in the co-occurrent network at time frame, *t*. This value is computed using the association strength formula described in Section 2.4.2.
- *role*_*relevance* is the value of the relevance for the specified *role*_*name* at time frame, *t*.

This formula takes into account not only the role relevance, which remains consistent regardless of the starting node, but also the relationship that nodes have with the central term used for the analysis. We computed the node relevance for all neighbors, considering each role and time frame. The resulting relevancies were then ranked in descending order, selecting the top 20 nodes. For example, Table 2 shows the results for “SARS-CoV-2” across 3 months. In role 0, the same key terms appear at the top almost in all the time frames. Role 2 may also reveal relevant aspects related to the virus. Role 2 shows great stability, which means that the terms here are constantly relevant during the evolution of researchers understanding. The top 20 list includes frequently occurring terms such as ACE2, serology (once again), MERS-CoV, spike protein, and transmission. ACE2, for instance, functions as the cellular receptor for SARS-CoV-2, while spike protein serves as the viral attachment protein. The analysis of tags in role 2 sheds light on the primary topics associated with infection and transmission mechanisms, testing, and treatments. These results, extracted without human refinement, are highly relevant and provide valuable insights into the research domain.

**Table 2:** Top 20 SARS-CoV-2 neighbors' tags for each role in different snapshots.

**Role 0**			
**2020-04**	**2020-05**	**2020-06**	**2020-07**
COVID-19	COVID-19	COVID-19	COVID-19
Coronavirus	Coronavirus	Coronavirus	Coronavirus
RNA-dependent RNA polymerase	ACE2	Pandemic	Zoonoses
Pandemic	Viruses	Serology	Respiratory infections
2019-nCoV	Hydroxychloroquine	Inflammation	Viruses
One Health	Pandemic	ACE2	Pandemic
Ebola	Remdesivir	Public health	Inflammation
Epidemiology	Favipiravir	Screening	Pneumonia
Remdesivir	Tocilizumab	Epidemiology	Serology
Pneumonia	MERS-CoV	Spain	Temperature
Public health	Respiratory infections	Respiratory infections	Spike protein
Antivirals	Pneumonia	Children	Transmission
Viruses	Convalescent plasma	2019-nCoV	Dysgeusia
Respiratory infections	Baricitinib	Viruses	Hyposmia
Molecular docking	Lopinavir	MERS-CoV	Humidity
Outbreak	Interferon	Spike protein	Olfaction
Infectious disease	Epidemiology	Vaccine	Olfactory dysfunction
Ebola virus (EBOV)	ARDS	Hydroxychloroquine	ELISA
Infection	Public health	Transmission	PPE
MERS-CoV	Angiotensin-converting enzyme 2	Basic reproduction number	Anosmia
**Role 2**			
**2020-04**	**2020-05**	**2020-06**	**2020-07**
2019-nCoV	ACE2	MERS-CoV	ACE2
Ebola	2019-nCoV	Serology	Zoonoses
ACE2	MERS-CoV	2019-nCoV	2019-nCoV
Remdesivir	RAAS	ACE2	MERS-CoV
MERS-CoV	TMPRSS2	Screening	PCR
Bats	Lopinavir	Transmission	Evolution
Homology modeling	Spike protein	Epidemiology	Obesity
Antiviral	Favipiravir	Inflammation	Inflammation
One Health	Serology	Pneumonia	Transmission
Drug repurposing	Zoonoses	Children	Viruses
Screening	Baricitinib	Remdesivir	Gene expression
**Role 0**			
**2020-04**	**2020-05**	**2020-06**	**2020-07**
Diagnosis	Hydroxychloroquine	Vaccine	Pneumonia
Molecular docking	Remdesivir	Treatment	Screening
Pathogenesis	Nucleocapsid protein	Fever	Serology
Serology	Children	Animal models	Data sharing
Spike protein	Clinical trials	TMPRSS2	Innate immunity
Wuhan	China	CRISPR	Respiratory infections
Pneumonia	Inflammation	Anosmia	Angiotensin-converting enzyme 2
Hydroxy chloroquine	Infection	Case fatality rate	China
Treatment	Wuhan	drug repurposing	Outbreak
**Role 4**			
**2020-04**	**2020-05**	**2020-06**	**2020-07**
Viral load	IgG	Drug repurposing	Metabolomics
Tocilizumab	Viral load	Saliva	Saliva
Remdesivir	Morbidity	TMPRSS2	Antigen
ACE2 receptor	Receptor binding domain	Liver injury	Molecular dynamics
Molecular dynamics	TMPRSS2	Animal models	Nanomedicine
ACE2	Pathogenesis	School	Immuno informatics
RT-PCR	Codon usage	Aspergillosis	TMPRSS2
ARB	High-flow nasal cannula	Newborn	Immunity
Molecular docking	Nucleocapsid protein	Viral load	qRT-PCR
Antibodies	COVID-19	nasopharyngeal swab	molecular docking
Pathogenesis	RT-PCR	Transmission potential	Swab
Homology modeling	Neurosurgery	Co-infection	Main protease
Antiviral	Baricitinib	Anosmia	Homology modeling
Therapeutics	Decontamination	Dengue	Viral load
Myocardial injury	Coagulopathy	Occupational health	Transplantation
Ground-glass opacities	Spike protein	Case fatality rate	Challenges
Anti-inflammatory	Hepatitis C virus	Main protease	Greece
Healthcare workers	ICU	Smell	Cardiac involvement
**Role 0**			
**2020-04**	**2020-05**	**2020-06**	**2020-07**
CT scan	MERS-CoV	ORF8	Pulmonary embolism (pe)
IL-6	N95 respirator	Survival	Vertical transmission

Role 4, another significant role in capturing relevant patterns, exhibits characteristics that are almost diametrically opposite to those of role 2. It can be described as a peninsula within the network structure, consisting of nodes located at the edges of the network (with low betweenness centrality) and connected to less relevant nodes (due to lower page rank values). In the list of the 20 most frequent elements, we find terms such as TMPRSS2, viral load, RT-PCR, nucleocapsid protein, and ORF8. For instance, nucleocapsid protein has been a target for serologic testing and has been considered at various stages in the development of a vaccine. It transitions across roles 0, 2, and 4, indicating changes in its relevance and the corresponding research focus over time, depending on experimental results and priorities. While nodes representing tags on these significant peninsulas are interesting, the true value lies in terms that consistently transition from role 4 to role 2, or even better, role 0. In an ideal scenario, we would observe terms that transition from consistently being in role 4, on the periphery of research, to consistently being in role 2, indicating their increased importance. This pattern signifies that certain approaches or techniques have proven their value and become dominant in the field. Conversely, when a tag transitions from roles 0 and 2 to role 4, or worse, to roles 1 or 3, it suggests that the associated research has been discarded or deprioritized. To conduct this analysis in a straightforward manner, we considered terms that consistently appear in role 4 (with a frequency higher than 2) and in at least one other role, specifically role 0 or 2. Table 3 presents some of these terms along with a brief explanation of their role in the research surrounding the virus.

**Table 3:** Some tags that consistently appear in role 4 and later in role 0 or role 2, indicating a change in relevancy of the tag over time.

**Tag**	**Comment**
Immunity	Non-specific immunity to fight infection. First line of defense against pathogens.
Main protease	Viral protease (3CLpro) required for processing viral proteins involved in virus replication.
MERS-CoV	Middle East Respiratory Syndrome CoV. Sarbecovirus related to SARS CoV-1 and SARS CoV-2.
Molecular docking	Process of computationally inserting small molecules into known structures of proteins.
Nucleocapsid protein	Viral protein that coats the viral genome to protect the nucleic acid.
RBD	Receptor binding domain. ~400 amino acid segment of SARS CoV-2 Spike responsible for binding to ACE2.
Remdesivir	Direct-acting antiviral originally developed for Ebola virus that targets the RdRp.
RNA-dependent RNA polymerase (RdRp)	RNA-dependent RNA polymerase. Viral RNA polymerase essential for viral transcription and genome replication. Druggable target.
RT-PCR	Method of amplifying DNA used for detection of viral genomes. RT denotes use of reverse transcriptase to convert viral RNA to DNA.
Seroprevalence	Prevalence of people positive for SARS CoV-2 serology.

Finally, it is intriguing to observe that conducting the same analysis on other tags provides a similar narrative but from different perspectives. Table 4 presents the most frequent tags resulting from the neighborhood analysis for COVID-19, which specifically represents the disease resulting from the infection of the SARS-CoV-2 virus. Role 0 and 2 shed light on aspects such as public and mental health, lockdown measures, and healthcare workers. On the other hand, role 4 reveals tags related to computed tomography (CT) scans, pneumothorax, and autopsy. Since the analysis is now centered on COVID-19, which represents the disease rather than the virus itself, the focus shifts toward treatments and their impact on individuals and public health, including mental health. Therefore, our TagGraph approach can support multiple narratives depending on the focal point of the analysis. Interestingly, these results align with most of the topics and questions that emerged from our survey of medical professionals, which will be discussed in the subsequent section.

**Table 4:** Frequency of the top 10 COVID-19 neighbors' tags across all the snapshots.

**Role 0**		**Role 2**		**Role 4**	
**Tag**	**Frequency**	**Tag**	**Frequency**	**Tag**	**Frequency**
SARS-CoV-2	21	Mortality	19	CT scan	5
Coronavirus	20	Social distancing	17	Response	4
Pandemic	20	Healthcare workers	17	PTSD	3
Public health	20	Lockdown	17	Pneumothorax	3
Inflammation	19	Anxiety	16	CT	3
Respiratory infections	15	Psychological distress	15	Practice	3
epidemiology	14	Mental health	14	Autopsy	3
Mental health	14	Pneumonia	13	Hematology (incl blood transfusion)	3
Viruses	13	ACE2	13	Anosmia	3
Telemedicine	12	Stress	12	Radiotherapy	2

### 3.3. Establishing the critical need for KGs in pandemic response: a qualitative analysis of clinicians' resources and knowledge gathering of COVID-19

To shed light on the critical needs that our approach aims to address, we conducted a qualitative study involving clinicians and researchers. The objective of this study was to gain a deeper understanding of the key information that could have guided and improved their early comprehension of COVID-19. Through this survey, we identified significant scientific “landmarks” that served as the foundation for building, testing, and validating our algorithms. By comprehending the cognitive models employed by the broader scientific community, we were better equipped to translate them into computational models using publicly available data. This, in turn, provides a platform for the rapid identification of coherent patterns within the scientific literature, thereby enhancing our ability to detect and respond to future pandemics and infectious outbreaks effectively.

Twenty-six clinicians (self-identifying as a physician, nurse, or other health professional) and research scientists (Ph.D. level) consented to participate in our survey (USF IRB Study #01211). Participants were recruited through e-mail, online message boards, and the web. Most survey respondents currently practice or work in the United States (73%), with others in Thailand (8%), Bangladesh (8%), the United Kingdom of Great Britain and Northern Ireland (4%), and locations undisclosed (7%). Participants were informed at the beginning of the survey that they could close their browser to discontinue or withdraw without penalty at any time. They were provided details about the survey, including its purpose to gather their perspectives on pieces of information that would have been helpful in combating the virus if known earlier, sources of information utilized by the scientific community, cognitive maps used by scientists to connect pieces of information, unresolved questions surrounding COVID-19, and seminal research findings on COVID-19. Our survey asked five open-response questions, including:

1. What do you know now that you wish you knew when COVID-19 first became a pandemic four months ago? For example, risk factors, spreading paths?
2. What sources of knowledge do you usually go to get your information on COVID-19? For example, clinicians, news, PUBMED, etc.?
3. How would you connect the different pieces of information together, or what components of the data would you have liked to have connected but wasn't connected before (even from multiple datasets)? For example, drug to molecular target, risk factors to symptom severity, geographic location to symptomology?
4. What do you consider to be the most critical unknown in COVID-19 that remains unresolved given the current research?
5. Which piece of research (please provide citation or PMID) do you consider to be seminal or “game changing” in shaping our current understanding of the virus? Please also provide the conceptual/empirical outcome that makes this research critical, for example, treatments, vaccines, pathways.

Three researchers from the project team coded the participant responses for questions 1−4 to identify prominent, recurring themes (Table 5). This process, which is a part of thematic analysis in qualitative research, represents the thorough evaluation of each participant's response to provide a word or phrase, called a code, that succinctly captures the core insight or meaning of that response. To consolidate the results of the survey, we performed an analysis on the concordance of the coding of responses by the three raters. The raters had a bi-rater agreement of 0.72, showing that two out of three raters agreed 72% of the time with the codes individually assigned across all participant responses. However, the consensus score across the three raters was poor at 32%. This lower level of agreement can be attributed to the larger number of codes available for selection (70) and the ability of the raters to assign a single response with up to six codes, creating more room for disagreement.

**Table 5:** Codes associated with survey questions one through four with their total frequency (in percentage) across all responses per question according to three raters.

**Q1: What do you know now that you wish you knew when COVID-19 first became a pandemic 4 months ago?**	**Q2: What sources of knowledge do you usually go to get your information on COVID-19?**	**Q3: How would you connect different pieces of information together, or what components of the data would you have liked to have connected?**	**Q4: What do you consider to be the most critical unknown in COVID-19 that remains unresolved given the current research?**
Transmission (20.4)	News (14.9)	Risk factors-severity (19.3)	Immunity (22.2)
Asymptomatic spread (15.7)	PUBMED (12.0)	Geography-infections (8.4)	Prevention (12.2)
PPE (13.9)	Broad (8.0)	Self (the participant would connect information on their own, using their own expertise) (7.2)	Successful treatments (8.9)
Risk factors (11.1)	CDC (6.9)	Geography-symptoms (6.0)	Reinfection severity (6.7)
Pandemic response (8.3)	Clinicians (6.9)	Clinical-virological (4.8)	Symptom effects (6.7)
Treatments (6.5)	NEJM (6.3)	Patient history-treatment response (4.8)	Long-term effects (6.7)
Viral dynamics (5.6)	State DOH (6.3)	Transmission-mortality (4.8)	Detailed pathophysiology (6.7)
Pre-symptomatic spread (4.6)	Colleagues (5.7)	Risk factors-demographics (3.6)	Infectious period (5.6)
Social distancing (4.6)	IDSA (5.1)	Social status-severity (3.6)	Government strategy (5.6)
Coagulopathy (3.7)	Google (5.1)	Hospitalizations-cases (3.6)	Infection rate (4.4)
Environmental susceptibility (3.7)	Universities (4.6)	Tests-positivity (3.6)	Symptom onset (3.3)
Antiviral susceptibility (1.9)	JAMA Network (3.4)	Behaviors-transmission (3.6)	Official transmission (3.3)
	SHEA (3.4)	Transmission-illness (3.6)	Testing accuracy (3.3)
	Lancet (2.9)	Viral structure-transmission (3.6)	Asymptomatic infections (2.2)
	WHO (1.7)	Various data sources (3.6)	cause of infection severity (2.2)
	No pre-prints (1.7)	Geography-mortality (2.4)	
	MMWR (1.1)	Social status-morality (2.4)	
	Social media (1.1)	Environment-transmission (2.4)	
	MedScape (1.1)	Research data sharing (2.4)	
	Promed (1.1)	Clinical data sharing (2.4)	
	Pre-prints (0.6)	Primary data access (2.4)	
		Countermeasures -transmission (1.2)	

High-level insights from our survey show that modes of transmission, particularly the infectivity of asymptomatic persons, were particularly concerning. Over half (67%) of respondents referred to the spread of the virus to some degree as information they wish they knew at the onset of the pandemic. One participant stated “*The role of asymptomatic transmission, the full role of respiratory transmission*” as information they wish knew. Similarly, participant 18 stated “*Risk factors, transmission of virus by people in different age groups, importance of wearing masks to reduce transmission*” as desired information at the onset of the virus. Others expressed concern regarding governmental responses to this and prior pandemics (e.g., its impact on job opportunities and the robustness of national policies), in addition to health-related vulnerabilities due to age.

Over half (54%) of respondents indicated PubMed, news sources (e.g., New York Times), unspecified peer-reviewed academic journals, other clinicians, Infectious Diseases Society of America (IDSA), and the United States Centers for Disease Control and Prevention (CDC) as major sources of information on COVID-19. Fewer mentioned sources included virtual seminars and meetings, sites which report local COVID-19 statistics, The New England Journal of Medicine (NEJM), and The Journal of the American Medical Association (JAMA). We note that this survey was conducted prior to the KG generation and analysis to both guide and verify the outcomes of these analyses; as such, PubMed from CORD-19 was used in the KG generation. At the time of the survey, most respondents had yet to connect information found at these sources, relying on publicly available data to correlate geographical location with virus spread, vulnerable populations, symptomology, and symptom severity. Many were curious about the connections between “*risk factors for symptom severity and levels of public adherence to personal protective equipment use protocols*,” and patient characteristics with positive or negative responses to treatments. Others noted a desire for improved coordination between countries, zip codes, and clinical trials, and felt public health interventions and preventative measures (e.g., vaccines), long-term immunity, data on prior infections, symptom onset and severity, and long-term complications as critical unknowns.

Nearly a third (30%) of respondents had yet to find what they would consider a seminal source of data that could shape our understanding of the virus. We refer the reader to sources that were provided at the following references: Sheahan et al. (2017), Andersen et al. (2020), Baum et al. (2020), Davies et al. (2020), The RECOVERY Collaborative Group (2020), He et al. (2020), Mehta et al. (2020), Nishiura et al. (2020), Shang et al. (2020), Wrapp et al. (2020), and Zost et al. (2020). We note that no sources were duplicated among responses. In summary, these results, such as a heavy reliance on news for data gathering and the lack of a seminal reference source that could have propelled scientific discovery regarding COVID-19, highlight a critical need for two important resources—an automated methodology for identifying emerging trends and knowledge concerning rapidly developing global diseases, and expedited consolidation and release of information in an easily digestible format.

## 4. Discussion

This article presents our temporal analysis conducted on the TagGraph, a knowledge graph generated by incorporating author-provided tags or keywords from scholarly articles. The purpose of this analysis is to facilitate temporal graph analysis for the exploration and comprehension of textual documents related to diseases. It is important to note that the TagGraph represents only a small portion of a larger knowledge graph that we have constructed for future investigations.

Our study highlights the significance of dynamic graph analysis, which provides roles and relevancies, and neighborhood analysis, which involves considerations of frequency and intersections. These analytical approaches enable the identification of patterns that can be easily described and understood. The primary achievement of our efforts lies in the ability to combine multiple complex analyses on a temporal knowledge graph and provide evidence and patterns that can be articulated in natural language, making them accessible to a wider audience. Furthermore, since the results can be generated without human intervention, the proposed approach can be automated and applied to various research topics and different disease outbreaks.

While our initial results are promising, there are numerous potential research avenues to explore. From an analysis perspective, there is room for enhancing the tags cleanup and merging process by testing alternative clustering algorithms and integrating ontologies, taxonomies, and dictionaries. These techniques, when combined, can result in a more refined set of initial tags, merging synonyms appropriately, and removing noisy and irrelevant tags. Furthermore, the proposed approach can be extended to other areas of the knowledge graph we have constructed, such as named entities that are automatically recognized. By applying the same methodology to these entities, we can uncover additional insights and patterns. Additionally, there are opportunities to explore alternative techniques for determining the number of roles and for factorization. By employing different approaches, we can better isolate interconnected patterns that would facilitate a clearer understanding of each role within the context of the tag knowledge graph, enhancing the communicative power of the results. Moreover, it would be worthwhile to investigate a deep learning-based approach to temporal graph analysis, as suggested by Rossi et al. (2020). Leveraging the capabilities of deep learning models could provide further advancements in understanding temporal dynamics and patterns within the knowledge graph.

From a data source point of view, there is an entire set of unexplored sources related to filed patents describing, for example, vaccines or procedures, that are not captured in our results. Other relevant sources are user-generated content in social networks or blog posts (Twitter, Facebook, Tumblr, etc.), news, country regulations and guidelines, public WHO, and other healthcare-related communication. These sources can provide other perspectives on the disease outbreak; patents can reveal the most valuable research results, public communication and country regulations can provide information about treatments best practices, or behavior, and social networks can provide people sentiment and general understanding. These research directions have the potential to enhance the effectiveness and interpretability of our approach, expanding its applicability to a broader range of domains and further improving the communication of valuable insights derived from temporal graph analysis.

## Data availability statement

This project uses existing, publicly available datasets. Existing datasets are available in publicly accessible repositories. These data can be found here: Hetionet (https://het.io/); Uniprot (https://www.uniprot.org/); CORD-19 (https://allenai.org/data/cord-19); Drug Repurposing Knowledge Graph (https://github.com/gnn4dr/DRKG); Gene Ontology (http://geneontology.org/); Medical Subject Headings (https://www.ncbi.nlm.nih.gov/mesh/).

## Ethics statement

The study involving human participants was approved by the IRB of the University of South Florida (USF IRB Study #01211). Written informed consent for participation in the study was provided by the participants.

## Author contributions

ST and AN contributed to conception and design of the study. AN and FM developed and analyzed the TagGraph approach. ST, TN, and MT reviewed and provided feedback on the results. SK and RK conducted the qualitative survey and analyzed these data presented in Section 3.3. TN and MT contributed to the manuscript revisions. All authors contributed to reading and approved the submitted version.

## References

[B1] Andersen K. G. Rambaut A. Lipkin W. I. Holmes E. C. Garry R. F. (2020). The proximal origin of SARS-CoV-2. Nat. Med. 26, 450–452. 10.1038/s41591-020-0820-9 32284615 PMC7095063

[B2] Bateman A. Martin M. J. Orchard S. Magrane M. Ahmad S. Alpi E. . (2022). UniProt: the universal protein knowledgebase in 2023. Nucleic Acids Res. 51, D523–D531. 10.1093/nar/gkac1052 36408920 PMC9825514

[B3] Baum A. Fulton B. O. Wloga E. Copin R. Pascal K. E. Russo V. . (2020). Antibody cocktail to SARS-CoV-2 spike protein prevents rapid mutational escape seen with individual antibodies. Science. 369, 1014–1018. 10.1126/science.abd0831 32540904 PMC7299283

[B4] Bults M. Beaujean D. J. de Zwart O. Kok G. van Empelen P. van Steenbergen J. E. . (2011). Perceived risk, anxiety, and behavioural responses of the general public during the early phase of the Influenza A (H1N1) pandemic in the Netherlands: results of three consecutive online surveys. BMC Public Health. 11, 1–13. 10.1186/1471-2458-11-2 21199571 PMC3091536

[B5] Canals A. (2005). “Knowledge diffusion and complex networks: a model of high-tech geographical industrial clusters,” in Proceedings of the 6th Europeanconference on organizational knowledge, Learning, and Capabilities (Waltham, MA: University of Warwick), 1–21.

[B6] Centers for Disease Control and Prevention (n.d.). Centers for Disease Control and Prevention. Available online at: https://data.cdc.gov/browse?tags=covid-19 (accessed January 10, 2023).

[B7] Cernile G. Heritage T. Sebire N. J. Gordon B. Schwering T. Kazemlou S. . (2021). Network graph representation of COVID-19 scientific publications to aid knowledge discovery. BMJ. 28, 100254. 10.1136/bmjhci-2020-100254 33419870 PMC7798427

[B8] Chan A. K. Nickson C. P. Rudolph J. W. Lee A. Joynt G. M. (2020). Social media for rapid knowledge dissemination: early experience from the COVID-19 pandemic. Anaesthesia. 75, 1579–1582. 10.1111/anae.15057 32227594 PMC7228334

[B9] Chen C. Ross K. E. Gavali S. Cowart J. E. Wu C. H. (2021). COVID-19 Knowledge Graph from semantic integration of biomedical literature and databases. Bioinformatics. 37, 4597–4598. 10.1093/bioinformatics/btab694 34613368 PMC8513397

[B10] Choi E. Bahadori M. T. Song L. Stewart W. F. Sun J. (2017). “August. GRAM: graph-based attention model for healthcare representation learning,” in Proceedings of the 23rd ACM SIGKDD International Conference on Knowledge Discovery and Data Mining, 787–795. 10.1145/3097983.3098126 33717639 PMC7954122

[B11] Choudhury N. Faisal F. Khushi M. (2020). Mining temporal evolution of knowledge graphs and genealogical features for literature-based discovery prediction. J. Informetr. 14, 101057. 10.1016/j.joi.2020.101057

[B12] Clements J. M. (2020). Knowledge and behaviors toward COVID-19 among US residents during the early days of the pandemic: cross-sectional online questionnaire. JMIR Public Health Surveill. 6, p.e19161. 10.2196/19161 32369759 PMC7212816

[B13] Cohan A. Feldman S. Beltagy I. Downey D. Weld D. S. (2020). “SPECTER: Document-level representation learning using citation-informed transformers,” in Proceedings of the 58th Annual Meeting of the Association for Computational Linguistics (Association for Computational Linguistics), 2270–2282. Available online at: https://aclanthology.org/2020.acl-main.207

[B14] Davies N. G. Klepac P. Liu Y. Prem K. Jit M. Eggo R. M. (2020). Age-dependent effects in the transmission and control of COVID-19 epidemics. Nat. Med. 26, 1205–1211. 10.1038/s41591-020-0962-9 32546824

[B15] Eck N. J. Waltman L. (2009). How to normalize cooccurrence data? An analysis of some well-known similarity measures. J. Assoc. Inf. Sci. Technol. 60, 1635–1651. 10.1002/asi.21075

[B16] Ester M. Kriegel H.-P. Sander J. Xu X. (1996). “A Density-Based Algorithm for Discovering Clusters in Large Spatial Databases with Noise,” in Proceedings of the Second International Conference on Knowledge Discovery and Data Mining (Palo Alto, CA: AAAI Press), 226–231.

[B17] Feng F. Tang F. Gao Y. Zhu D. Li T. Yang S. . (2022). GenomicKB: a knowledge graph for the human genome. Nucleic Acids Res. 51, D950–D956. 10.1093/nar/gkac957 36318240 PMC9825430

[B18] Gene Ontology Consortium (2004). The Gene Ontology (GO) database and informatics resource. Nucleic Acids Res. 32, D258–D261. 10.1093/nar/gkh036 14681407 PMC308770

[B19] Govindapillai S. Soon L. K. Haw S. C. (2021). An empirical study on Resource Description Framework reification for trustworthiness in knowledge graphs. F1000Research. 10, 2. 10.12688/f1000research.72843.2 34900233 PMC8634049

[B20] Grishman R. (2015). Information extraction. IEEE Intell. Syst. 30, 8–15. 10.1109/MIS.2015.68

[B21] Grishman R. Sundheim B. (1996). “Message understanding conference- 6: A brief history,” in COLING 1996 Volume 1: The 16th International Conference on Computational Linguistics. Available online at: https://aclanthology.org/C96-1079

[B22] He X. Lau E. H. Wu P. Deng X. Wang J. Hao X. . (2020). Temporal dynamics in viral shedding and transmissibility of COVID-19. Nat. Med. 26, 672–675. 10.1038/s41591-020-0869-5 32296168

[B23] Henderson K. Gallagher B. Eliassi-Rad T. Tong H. Basu S. Akoglu L. . (2012). “RolX: structural role extraction & mining in large graphs,” in Proceedings of the 18th ACM SIGKDD International Conference on Knowledge Discovery and Data Mining, 1231–1239.

[B24] Henderson K. Gallagher B. Li L. Akoglu L. Eliassi-Rad T. Tong H. . (2011). “It's who you know: graph mining using recursive structural features,” in Proceedings of the 17th ACM SIGKDD International Conference on Knowledge Discovery and Data Mining, 663–671.

[B25] Himmelstein D. S. Lizee A. Hessler C. Brueggeman L. Chen S. L. Hadley D. . (2017). Systematic integration of biomedical knowledge prioritizes drugs for repurposing. Elife. 6, e26726. 10.7554/eLife.26726.017 28936969 PMC5640425

[B26] Hirschberg J. Manning C. D. (2015). Advances in natural language processing. Science. 349, 261–266. 10.1126/science.aaa8685 26185244

[B27] Hogan A. Blomqvist E. Cochez M. d'Amato C. Melo G. D. Gutierrez C. . (2021). Knowledge graphs. ACM. 54, 1–37. 10.1145/3447772

[B28] Hyland B. Wood D. (2011). “The joy of data-a cookbook for publishing linked government data on the web,” in Linking Government Data. New York, NY: Springer New York, 3–26. 10.1007/978-1-4614-1767-5_1

[B29] Ioannidis V. N. Song X. Manchanda S. Li M. Pan X. Zheng D. . (2020). DRKG - Drug Repurposing Knowledge Graph for COVID-19. Available online at: https://github.com/gnn4dr/DRKG/ (accessed January 10, 2023).

[B30] Li F. L. Chen H. Xu G. Qiu T. Ji F. Zhang J. . (2020). “October. AliMeKG: Domain knowledge graph construction and application in e-commerce,” in Proceedings of the 29th ACM International Conference on Information & Knowledge Management, 2581–2588. 10.1145/3340531.3412685

[B31] Li Z. Zeng J. Chen Y. Liang Z. (2022). “AttacKG: constructing technique knowledge graph from cyber threat intelligence reports,” in European Symposium on Research in Computer Security. Cham: Springer. 10.1007/978-3-031-17140-6_29

[B32] Lin Y. Huang L. Nie S. Liu Z. Yu H. Yan W. . (2011). Knowledge, attitudes and practices (KAP) related to the pandemic (H1N1) 2009 among Chinese general population: a telephone survey. BMC Infect. Dis. 11, 1–9. 10.1186/1471-2334-11-128 21575222 PMC3112099

[B33] Lipscomb C. E. (2000). Medical subject headings (MeSH). Bullet. Med. Library Assoc. (2000) 88, 265. PMC35238 10928714

[B34] Liu W. Zhou P. Zhao Z. Wang Z. Ju Q. Deng H. . (2020). “K-bert: Enabling language representation with knowledge graph,” in Proceedings of the AAAI Conference on Artificial Intelligence, 2901–2908. 10.1609/aaai.v34i03.5681

[B35] Liu Y. Zeng Q. Ordieres Mer,é J. Yang H. (2019). Anticipating stock market of the renowned companies: a knowledge graph approach. Complexity. 2019, 9202457 10.1155/2019/9202457

[B36] Luan Y. He L. Ostendorf M. Hajishirzi H. (2018). “Multi-task identification of entities, relations, and conference for scientific knowledge graph construction,” in Proceedings of the 2018 Conference on Empirical Methods in Natural Language Processing (Brussels: Association for Computational Linguistics), 3219–3232. Available online at: https://aclanthology.org/D18-1360

[B37] Mehta P. McAuley D. F. Brown M. Sanchez E. Tattersall R. S. Manson J. J. (2020). COVID-19: consider cytokine storm syndromes and immunosuppression. Lancet. 395, 1033–1034. 10.1016/S0140-6736(20)30628-0 32192578 PMC7270045

[B38] Michel F. Gandon F. Ah-Kane V. Bobasheva A. Cabrio E. Corby O. . (2020). “Covid-on-the-Web: Knowledge graph and services to advance COVID-19 research. In The Semantic Web–ISWC 2020:19th International Semantic Web Conference, Athens, Greece, November 2–6, 2020,” in Proceedings, Part II 19. Cham: Springer International Publishing, 294–310. 10.1007/978-3-030-62466-8_19

[B39] Montemurro M. A. Zanette D. H. (2013). Keywords and co-occurrence patterns in the voynich manuscript: an information-theoretic analysis. PLoS ONE. 8, e66344. 10.1371/journal.pone.0066344 23805215 PMC3689824

[B40] Negro A. (2021). Graph-Powered Machine Learning. Shelter Island, NY: Manning Publications.

[B41] Negro A. Kus V. Futia G. Montagna F. (2023). Knowledge Graph Applied. Shelter Island, NY: Manning Publications (MEAP).

[B42] Ngomo A. C. N. Auer S. Lehmann J. Zaveri A. (2014). “Introduction to linked data and its lifecycle on the web,” in Reasoning Web. Reasoning on the Web in the Big Data Era. Reasoning Web 2014. Lecture Notes in Computer Science, Vol. 8714, eds M. Koubarakis, G. Stamou, G. Stoilos, I. Horrocks, P. Kolaitis, G. Lausen, and G. Weikum (Cham: Springer). 10.1007/978-3-319-10587-1_1

[B43] Nishiura H. Oshitani H. Kobayashi T. Saito T. Sunagawa T. Matsui T. . (2020). Closed environments facilitate secondary transmission of coronavirus disease 2019 (COVID-19). medRxiv [Preprint]. 10.1101/2020.02.28.20029272

[B44] Pan R. K. Sinha S. Kaski K. Saramäki J. (2012). The evolution of interdisciplinarity in physics research. Sci. Rep. 2, 551. 10.1038/srep00551 22870380 PMC3412321

[B45] Purohit S. Van N. Chin G. (2021). “December. Semantic property graph for scalable knowledge graph analytics,” in 2021 IEEE International Conference on Big Data (Big Data). Orlando, FL: IEEE, 2672–2677. 10.1109/BigData52589.2021.9671547

[B46] Rossi E. Chamberlain B. Frasca F. Eynard D. Monti F. Bronstein M. (2020). Temporal graph networks for deep learning on dynamic graphs. arXiv. 10.48550/arXiv.2006.10637

[B47] Rossi R. Gallagher B. Neville J. Henderson K. (2012). “Role-Dynamics: Fast Mining of Large Dynamic Networks,” in Proceedings of the 21st International Conference on World Wide Web, 997–1006.

[B48] Schulz S. Costa C. M. Kreuzthaler M. Minarro-Giménez J. A. Andersen U. Jensen A. B. . (2014). “Semantic relation discovery by using co-occurrence information,” in 4th Workshop on Building and Evaluating Resources for Health and Biomedical Text Processing (BioTxtM 2014), held at the Ninth International Conference on Language Resources and Evaluation (Reykjavik).

[B49] Shang J. Ye G. Shi K. Wan Y. Luo C. Aihara H. . (2020). Structural basis of receptor recognition by SARS-CoV-2. Nature. 581, 221–224. 10.1038/s41586-020-2179-y 32225175 PMC7328981

[B50] Sheahan T. P. Sims A. C. Graham R. L. Menachery V. D. Gralinski L. E. Case J. B. . (2017). Broad-spectrum antiviral GS-5734 inhibits both epidemic and zoonotic coronaviruses. Sci. Transl. Med. 9, eaal3653. 10.1126/scitranslmed.aal3653 28659436 PMC5567817

[B51] Shen Z. Zhang Y. H. Han K. Nandi A. K. Honig B. Huang D. S. (2017). miRNA-disease association prediction with collaborative matrix factorization. Complexity. 2017, 2498957. 10.1155/2017/2498957

[B52] Song M. Han N. G. Kim Y. H. Ding Y. Chambers T. (2013). Discovering implicit entity relation with the gene-citation-gene network. PLoS ONE. 8, e84639. 10.1371/journal.pone.0084639 24358368 PMC3866152

[B53] Su H.-N. Lee P. C. (2010). Mapping knowledge structure by keyword co-occurrence: a first look at journal papers in technology foresight. Scientometrics. 85, 65–79. 10.1007/s11192-010-0259-8

[B54] Szekely P. Knoblock C. A. Slepicka J. Philpot A. Singh A. Yin C. . (2015). “Building and using a knowledge graph to combat human trafficking,” in International Semantic Web Conference. Cham: Springer, 205–221. 10.1007/978-3-319-25010-6_12

[B55] The RECOVERY Collaborative Group (2020). Dexamethasone in hospitalized patients with COVID-19—preliminary report. N. Engl. J. Med. 84, 693–704. 10.1056/NEJMoa2021436 PMC7383595 32678530

[B56] Villazón-Terrazas B. Vilches-Blázquez L. M. Corcho O. Gómez-Pérez A. (2011). Methodological guidelines for publishing government linked data. Linking Gov. Data. 27–49. 10.1007/978-1-4614-1767-5_2

[B57] Wahltinez O. Cheung A. Alcantara R. Cheung D. Daswani M. Erlinger A. . (2022). COVID-19 Open-Data a Global-Scale Spatially Granular Meta-Dataset for Coronavirus Disease. Available online at: https://goo.gle/covid-19-open-data (accessed January 10, 2023). 10.1038/s41597-022-01263-z PMC9005692 35413965

[B59] Wang L. Lo K. Chandrasekhar Y. Reas R. Yang J. Burdick D. . (2020). CORD-19: The COVID-19 open research dataset. arXiv [Preprint]. arXiv: 2004.10706v4. 10.48550/arXiv.2004.1070632510522

[B60] Wang Q. Li M. Wang X. Parulian N. Han G. Ma J. . (2020). COVID-19 literature knowledge graph construction and drug repurposing report generation. arXiv. 10.18653/v1/2021.naacl-demos.836568019

[B61] Wise C. Vassilis N. Miguel Romero C. Xiang S. George P. Ninad K. . (2020). “COVID-19 knowledge graph: accelerating information retrieval and discovery for scientific literature,” in AACL-IJCNLP 2020 Workshop on Integrating Structured Knowledge and Neural Networks for NLP (KNLP).

[B62] Wrapp D. Wang N. Corbett K. S. Goldsmith J. A. Hsieh C. L. Abiona O. . (2020). Cryo-EM structure of the 2019-nCoV spike in the prefusion conformation. Science. 367, 1260–1263. 10.1126/science.abb2507 32075877 PMC7164637

[B63] Xu D. Ruan C. Korpeoglu E. Kumar S. Achan K. (2020). “Product knowledge graph embedding for e-commerce,” in Proceedings of the 13th International Conference on Web Search and Data Mining (672–680). 10.1145/3336191.3371778

[B64] Yang Y. Wu M. Cui L. (2011). Integration of three visualization methods based on co-word analysis. Scientometrics. 90, 659–673. 10.1007/s11192-011-0541-4

[B65] Zhou K. Zhao W. X. Bian S. Zhou Y. Wen J.-R. Yu J. (2020). “Improving conversational recommender systems via knowledge graph based semantic fusion,” in Proceedings of the 26th ACM SIGKDD International Conference on Knowledge Discovery & amp; Data Mining (KDD '20) (New York, NY: Association for Computing Machinery), 1006–1014. 10.1145/3394486.3403143

[B66] Zost S. J. Gilchuk P. Case J. B. Binshtein E. Chen R. E. Nkolola J. P. . (2020). Potently neutralizing and protective human antibodies against SARS-CoV-2. Nature. 584, 443–449. 10.1038/s41586-020-2548-6 32668443 PMC7584396

